# Prophetic values of lung ultrasound score on post-extubation distress in patients with acute respiratory distress syndrome

**DOI:** 10.1186/s40001-022-00652-9

**Published:** 2022-02-22

**Authors:** Ran Wang, Benquan Qi, Xiaohua Zhang, Liang Meng, Xiaofei Wu

**Affiliations:** Department of Emergency Internal Medicine, The First Affiliated Hospital of Bengbu Medical College, No. 287 Changhuai Road, Bengbu, 233004 Anhui China

**Keywords:** Lung ultrasonography (LUS), Acute respiratory distress syndrome (ARDS), Spontaneous breathing trial (SBT), Post-extubation distress

## Abstract

**Background:**

Acute respiratory distress syndrome (ARDS) has been a prevalent disease in ICU with mortality of up to 27–45%. A considerable proportion of extubated ARDS patients passing spontaneous breathing trial (SBT) still requires reintubation.

**Methods:**

Lung ultrasonography (LUS) was used to predict the success rate of extubation. Ninety-two patients passing the 60-min SBT were included in this prospective research. Their clinical characters including LUS, APACHE II, SOFA, CPIS, EVLWI and PaO_2_/FiO_2_ were collected before the SBT. Another two LUS assessments were performed at the end of and 4 h after SBT. LUS results were evaluated and scored by two independent experts, and the correlations of LUS scores, APACHE-II scores, SOFA scores, CPIS and EVLWI with the success rate of extubation and rate of reintubation were analyzed.

**Results:**

Failed weaning and reintubation of ARDS patients were correlated with higher LUS scores both before and after SBT. Post-extubation distress was correlated with higher APACHE-II scores, SOFA scores, CPIS and EVLWI before SBT. There were positive correlations between the LUS score and APACHE-II score, SOFA score, CPIS and EVLWI before SBT, respectively.

**Conclusion:**

LUS score measured at the end of 60-min SBT could be used to predict post-extubation distress in ARDS patients.

## Background

Acute respiratory distress syndrome (ARDS) is a complex syndrome characterized by acute progressive hypoxic respiratory failure post-lung capillary injury [1]. ARDS has been a prevalent disease in intensive care unit (ICU) [2]. Trauma or inflammatory mediators cause pulmonary vascular congestion, vascular endothelial cell damage, increased vascular permeability, and aggregation of inflammatory cells in the alveolar cavity [3]. Related inflammation factors and chest computed tomography (CT) are two important indicators for clinical evaluation of ARDS [4]. However, CT examination is frequently limited in critically ill patients with unstable hemodynamics or patients who cannot move due to assisted ventilation [5].

Ultrasonography began to be used in lung examinations in critically ill and emergency patients such as acute respiratory failure in the late 1990s [6, 7]. As Lichtenstein discovered the application value of lung ultrasonography (LUS) and established feasible operation and scoring rules, the imaging theory and clinical process of LUS have been established [8]. LUS can quickly differentiate and diagnose a variety of lung diseases, with increased diagnostic sensitivity and specificity [9]. LUS provides a lot of effective information for the alterations of lung morphology [10]. Indirect signs shown by ultrasound such as abnormal pleural line, disappearance of A-line, abnormal increase of B-line, lung consolidation and other manifestations can be used for the diagnosis of multiple diseases [11]. Previous studies have demonstrated that lung consolidation accompanied by bronchial inflation, abnormal pleural lines, diffuse pulmonary edema and disappearance of A-line are the main signs of LUS in ARDS patients [12]. Furthermore, pulmonary consolidation, diffuse pulmonary edema and abnormal pleural line are closely related to the onset of ARDS [13].

LUS has become an important method for clinical evaluation of patients’ condition. However, there is still insufficient research for LUS to guide and evaluate the success rate of extubation in ARDS patients. Among ARDS patients who are extubated after spontaneous breathing trial (SBT), a considerable proportion of patients may still need to be reintubated, which brings a serious burden to the patients’ health [14]. Therefore, we designed an evaluation system including LUS score combined with Clinical Pulmonary Infection Score (CPIS), Acute Physiology and Chronic Health Evaluation (APACHE II), Sequential Organ Failure assessment (SOFA), extravascular lung water indices (EVLWI) and SBT to assess the success rate and reintubation rate of ARDS patients in this study.

## Methods

### Study design

To investigate the correlation between the LUS scores and the incidence of post-extubation distress following SBT in ARDS patients, a prospective clinical trial was performed in this research. We evaluated the characteristics and extubating outcomes of ARDS patients at 48 h post-SBT. This study was approved by the Ethics Committee of the First Affiliated Hospital of Bengbu Medical College.

### Participants

The inclusion criteria were: (1) over 18 years old. (2) Participants who were admitted in ICU. (3) Patients who met the diagnostic standard of ARDS (The Berlin definition). Patients who met the following criteria were excluded from our research: (1) less than 18 years old. (2) Patients in pregnancy. (3) Patients with a history of chronic obstructive pulmonary disease, including pulmonary embolism, trauma, undrained pneumothorax, and interstitial lung disease. (4) Patients with severe cardiac insufficiency and acute coronary syndrome. (5) Patients with autoimmune diseases.

A total of 176 ARDS patients were recruited and assessed for the eligibility to our study. Among the patients, 47 participants were excluded because they did not meet the inclusion criteria and 21 patients refused to continue participating in the research. The remaining 108 patients were assessed for their clinical scores including LUS, APACHE II, SOFA, CPIS, EVLWI and PaO_2_/FiO_2_ before SBT. Ninety two of them passed the 60-min SBT and received the LUS at the end of and 4 h post the trial. 48 h after SBT, 61 patients ended up with successful weaning of respirator, while 31 patients exhibited extubation distress and needed to be reintubated.

### Spontaneous breathing trial

Patients who met all the following conditions were allowed to undergo SBT. (1) Patients whose condition improved or whose disease was cured. (2) Oxygenation index: PaO_2_/FiO_2_ ≥ 150 mmHg; PEEP ≤ 5 cmH_2_O; FiO_2_ ≤ 40%; pH ≥ 7.25; for COPD patients: pH > 7.30, FiO_2_ < 35%, PaO_2_ > 50 mm Hg. (3) Patients with stable hemodynamics, no dynamic changes of myocardial ischemia, and no clinically significant hypotension. (4) Patients with the ability to breathe spontaneously.

Low-level support and continuous positive pressure ventilation (≤ 5 cmH_2_O or PSV ≤ 7 cmH_2_O) were used as SBT for 60 min. The clinical indicators of SBT success were: (1) FiO_2_ < 40%, SpO_2_ ≥ 90%; PaO_2_ ≥ 60 mmHg; pH ≥ 7.32; PaCO_2_ increase ≤ 10 mmHg. (2) Stable hemodynamics: HR < 120 times/min; HR change < 20%; systolic blood pressure < 180 and > 90 mmHg; blood pressure changes < 20%. (3) Respiration RR ≤ 35 times/min; RR changes less than 50%.

### Lung ultrasonography

A senior physician was in charge of lung and ultrasound heart examination. Xario 100 ultrasound machine, phased array convex probe, and ultrasound probe (3.5–10.0 MHz) were used for the ultrasound examination in this study. A total of 12 lung areas on both sides of the front chest wall, side chest wall and back chest wall were examined and video data were recorded. The lungs were divided into three areas: anterior, lateral, and posterior by the parasternal line, anterior axillary line, and posterior axillary line. The left and right lungs were divided into 6 areas with the nipple plane as the upper and lower boundary. Two trained senior physicians separately scored the LUS results. Lung ultrasound images were defined as: (1) normal ventilation zone (N): lung sliding sign with A-line or less than 2 separate B-lines; (2) moderately reduced lung ventilation: multiple, typical B-line (B1 line); (3) severe lung reduced ventilation area: multiple fusion B line (B2-line); (4) consolidation lung area: tissue image with typical bronchial inflation. Each area was scored with the most severe performance: N was 0 points, B1 line was 1 point, B2 line was 2 points, and C was 3 points. The LUS score of one particular patient was the sum of the scores of each area, with 0–36 points.

### Extravascular lung water indices

The right subclavian vein of ARDS patients was catheterized and connected with a temperature sensor. The PiCCO catheter was inserted into the patient through the femoral artery, and the catheter electrode was connected to the PiCCO monitor. 10 ml of 0.9% sodium chloride at 0–8 °C was quickly injected into the blood vessel through a deep venous catheter. The injection was repeated for 3 times and the average EVLW score was recorded.

### Clinical characteristics

A senior doctor was responsible for collecting clinical data and clinical scores of patients upon admission, including baseline data, hemodynamic parameters, lactate, respiratory parameters, APACHE II, SOFA, CPIS, EVLWI assessment scores following the instructions of previous research before the SBT performance [15–18].

### Statistical analysis

The categorized variables were shown as frequency or percentage. Mean and standard deviation were utilized to demonstrate continuous variables. Chi-square test (or Fisher’s exact test) was used to compare the classification variables. One-way ANOVA and Tukey’s post hoc test were used to analyze the continuous variables, and contingency table Chi-square test was acquired for the comparison of outcomes among different groups. The SAS 17.0 software (SAS Institute Inc., Cary, NC, USA) was purchased for the statistical analysis in this study.

## Results

### Study design and participants

**Fig. 1:**
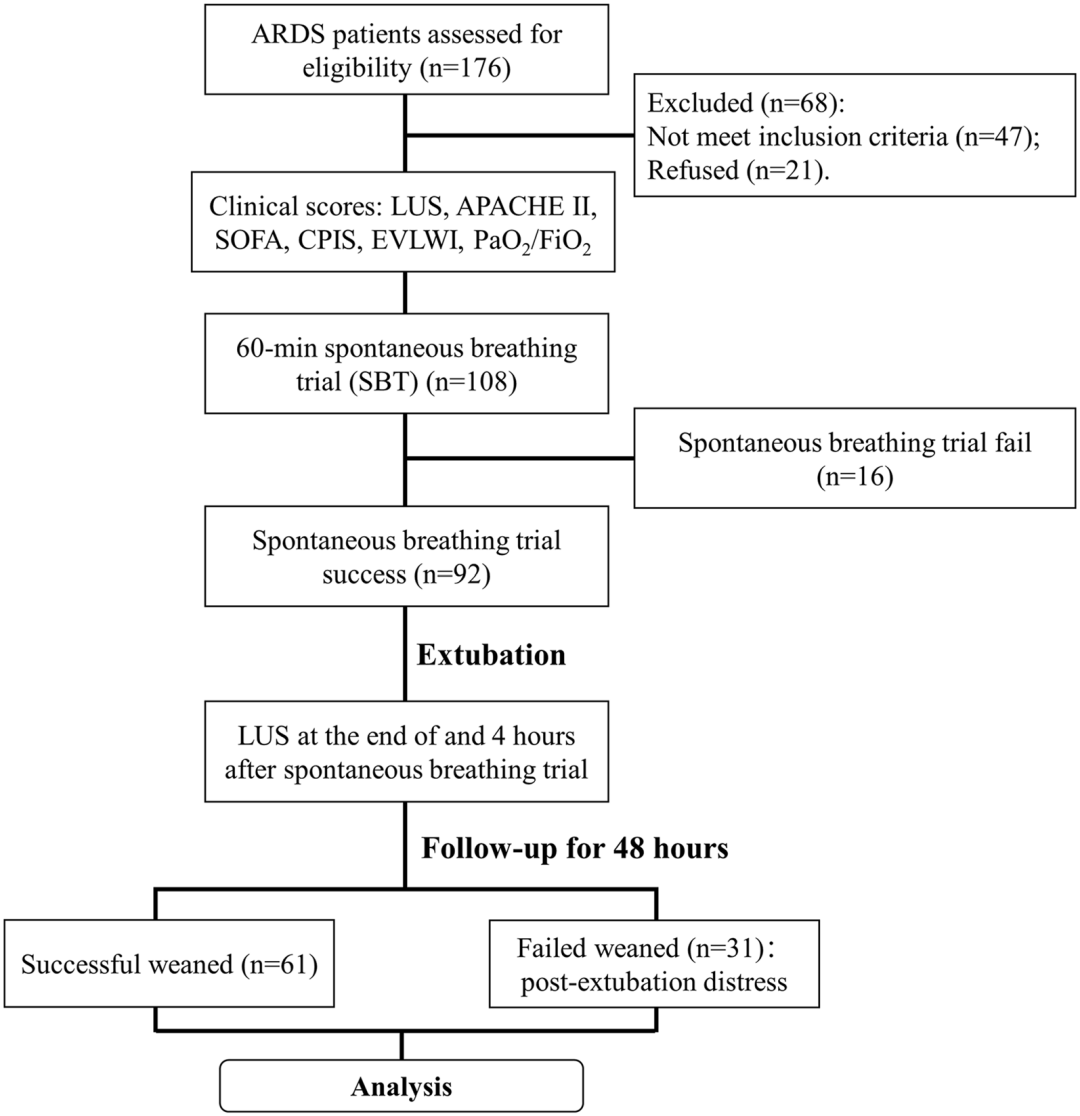
Experimental diagram in this study

To investigate the correlation between the LUS scores and the incidence of post-extubation distress following the 60-min SBT in ARDS patients, a retrospective trial was performed in this research. As shown in Fig. 1, a total of 176 ARDS patients were recruited and assessed for their eligibility to our study. Among all the patients, 47 participants were excluded because they did not meet the inclusion criteria and 21 patients refused to participate in this research. The clinical scores including LUS, APACHE II, SOFA, CPIS, EVLWI and PaO_2_/FiO_2_ of the rest 108 patients were tested before SBT. Ninety Two of them passed the 60-min SBT and received the LUS at the end of and 4 h post the trial. They were divided into the successfully weaned and failed weaned group according to their extubating outcomes at 48 h post-extubation. 48 h after SBT, 61 patients ended up with successful weaning of respirator, while 31 patients exhibited post-extubation distress and needed to be reintubated.

### Clinical characteristics of the study population

Characteristics of the study population are shown in Table 1. The demographic parameters collected from patients of the two groups included age, gender, etiology of ARDS, disease onset time, Berlin diagnostic criteria. As shown in Table 1, there were no significant differences in any of the above characteristics between participants in these two groups at the beginning of our research (*p* > 0.05).

**Table 1:** Demographic and clinical characteristics of participants analyzed

Variable	Study group		*p*
	Successfully weaned (*n* = 61)	Failed weaned (*n* = 31)	
Age (years)	60.25 ± 13.64	66.92 ± 14.53	0.174
Gender			
Male	33 (54.1%)	16 (51.6%)	0.829
Female	28 (45.9%)	15 (48.4%)	
Etiology of ARDS			
Pneumonia	29 (47.5%)	12 (38.7%)	0.779
Aspiration	9 (14.8%)	3 (9.7%)	
Sepsis	11 (18.0%)	8 (25.8%)	
Cardio-pulmonary resuscitation	5 (8.2%)	3 (9.7%)	
Trauma	7 (11.5%)	5 (16.1%)	
Onset			
≤ 48 h	43 (70.5%)	14 (45.2%)	0.023
> 48 h	18 (29.5%)	17 (54.8%)	
Berlin diagnostic criteria			
Mild	24 (39.3%)	6 (19.4%)	0.092
Moderate	18 (29.5%)	9 (29.0%)	
Severe	19 (31.2%)	16 (51.6%)	

Values are expressed as *n* (percentage, %) or mean ± SD. *p* values for each group were derived from either unpaired t test or Mann–Whitney test as appropriate. Chi-square test or Fisher’s exact test was used for assessing distribution of observations or phenomena between different groups

### Post-extubation distress was correlated with higher LUS scores before, at the end of and 4 h after SBT

**Fig. 2:**
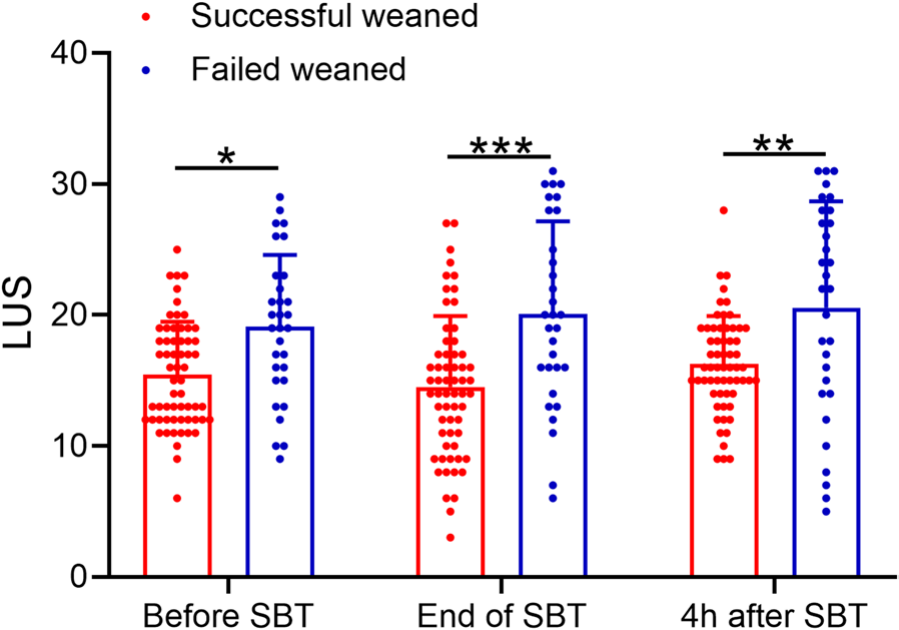
Changes in lung ultrasound scores (LUS) between successfully and failed weaned patients during the spontaneous breathing trial (SBT). Data were presented as mean ± SD showing all the data. Two-way ANOVA followed Sidak’s multiple comparisons test. *p < 0.05, **p < 0.01, ***p < 0.001

To demonstrate the correlation between LUS scores and the extubating outcomes of ARDS patients, the LUS scores tested before, at the end of and 4 h after SBT in the two groups are shown in Fig. 2. The LUS scores of patients with post-extubation distress 48 h after SBT were all significantly higher at the three time points than the successfully weaned group, indicating that there were significant differences in the LUS before and after SBT between the successful and unsuccessful extubation groups.

### Post-extubation distress was correlated with higher APACHE II, SOFA, CPIS, EVLWI tested before the SBT

**Fig. 3:**
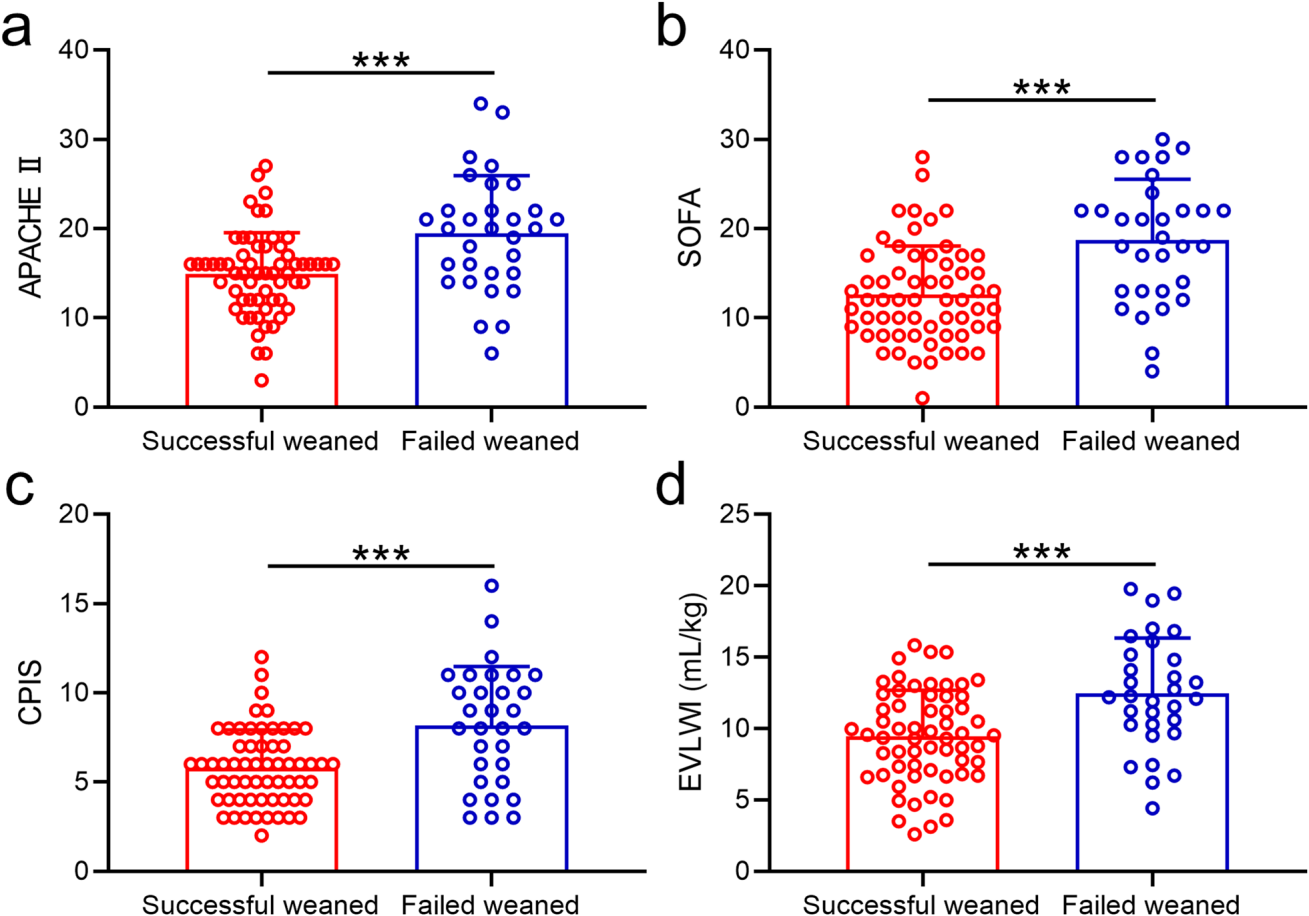
Acute physiology and chronic health evaluation II (APACHE II) scores (**a**), Sequential Organ Failure Assessment (SOFA) scores (**b**), clinical pulmonary infection score (CPIS) (**c**), and extravascular lung water indices (EVLWI) (**d**) measurements between successfully and failed weaned patients before the spontaneous breathing trial (SBT). Data were presented as mean ± SD showing all the data. One-way ANOVA followed by a Dunn’s multiple comparisons test. ***p < 0.001

To further identify the relationship between the post-extubation distress and the APACHE II, SOFA, CPIS, EVLWI scores, we measured them before the performance of SBT following the instructions of previous research. As shown in Fig. 3, the scores of APACHE II (Fig. 3A), SOFA (Fig. 3B), CPIS (Fig. 3C), EVLWI (Fig. 3D) in the failed weaned group were all statistically higher than the successfully weaned group. These results demonstrated that post-extubation distress was correlated with higher APACHE II, SOFA, CPIS, EVLWI scores, which was also consistent with previous discoveries.

### Spearman’s correlations between LUS and APACHE II, SOFA, CPIS and EVLWI scores before the SBT

**Fig. 4:**
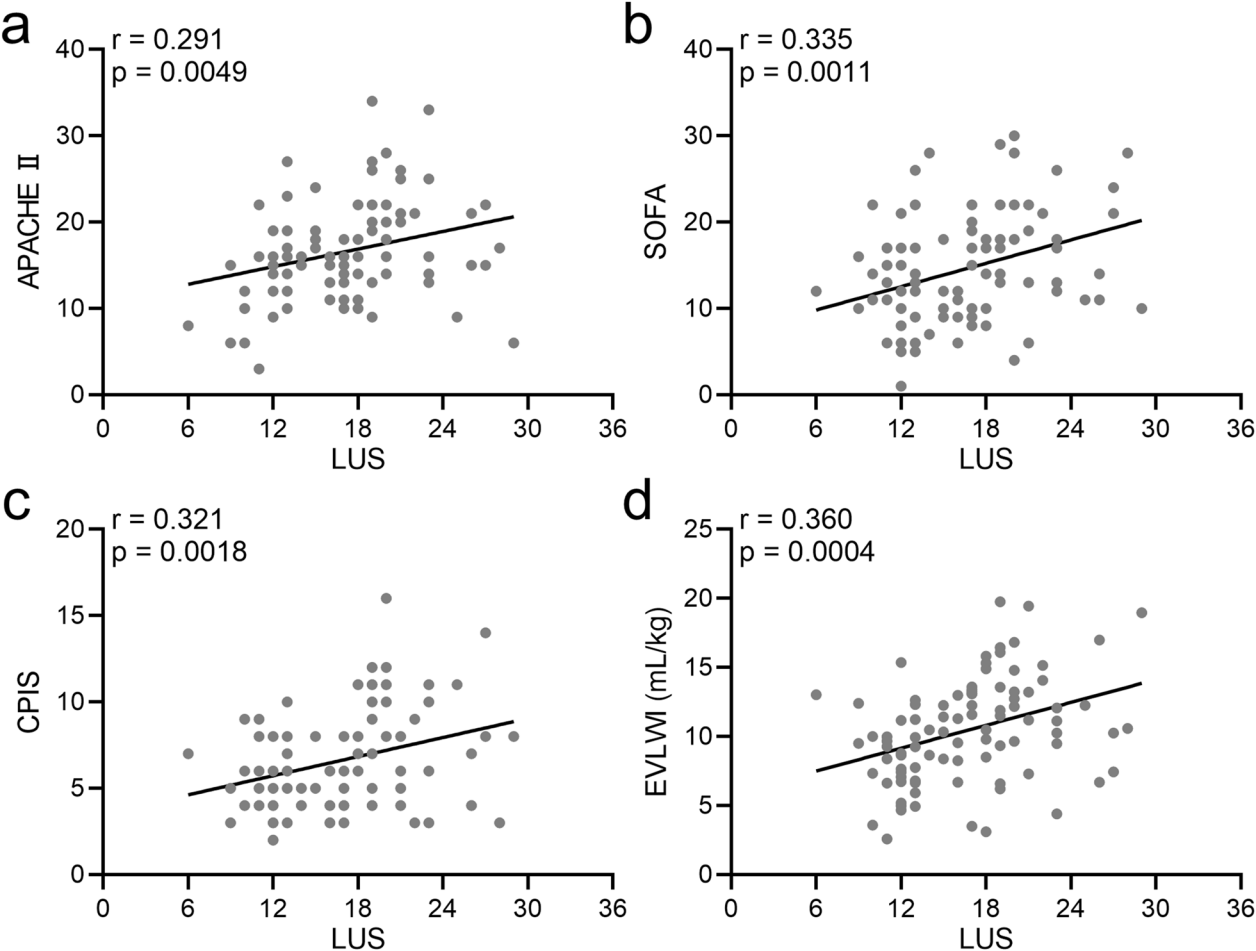
Spearman’s correlations between lung ultrasound scores (LUS) and acute physiology and chronic health evaluation II (APACHE II) scores (**a**), sequential organ failure assessment (SOFA) scores (**b**), clinical pulmonary infection score (CPIS) (**c**), and extravascular lung water indices (EVLWI) (**d**) in analyzed patients before the spontaneous breathing trial (SBT)

To investigate the correlation between LUS and APACHE II, SOFA, CPIS and EVLWI scores tested before the SBT, Spearman’s correlation analysis was performed. Significant positive liner correlations of LUS score with APACHE II, SOFA, CPIS and EVLWI scores are presented in Fig. 4A–D (*r* values were 0.291, 0.335, 0.321, 0.360, respectively, and all *p* < 0.001).

### ROC analysis of LUS scores measured at the end of SBT to predict post-extubation distress

**Fig. 5:**
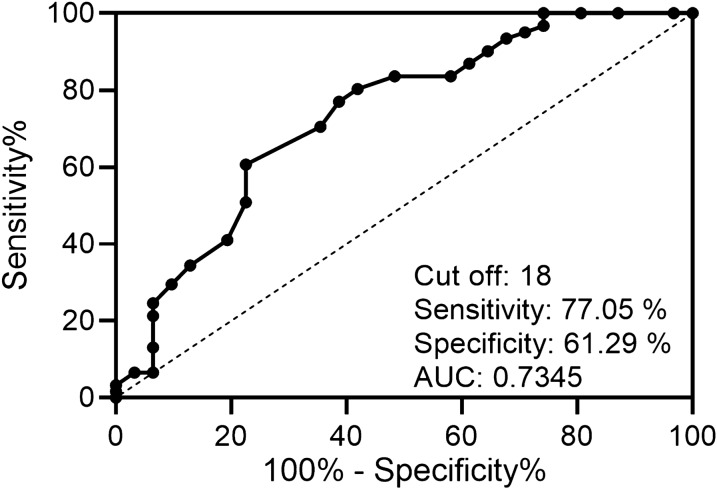
ROC analysis of lung ultrasound score (LUS) measured at the end of spontaneous breathing trial (SBT) to predict post-extubation distress

To demonstrate the predictive value of LUC scores tested at the end of SBT on the incidence of post-extubation distress of ARDS patients, receiver operating characteristic (ROS) curve was calculated. As presented in Fig. 5, a cut-off value of LUS score tested at the end of SBT was 18 and the corresponding area under curve (AUC) was 0.7345, with sensitivity 77.05% and specificity 61.29%, suggesting that LUC scores tested at the end of SBT exhibited potential to predict post-extubation distress.

## Discussion

ARDS is mainly manifested as pulmonary edema and hyaline membrane formation caused by diffuse damage and increased permeability of pulmonary capillaries [19]. As an acute respiratory failure syndrome, its clinical features mainly include respiratory distress and asphyxia [20]. ARDS is harmful for the health of patients. Although its incidence is relatively low, its fatality rate is extremely high [21]. There are many causes of ARDS, including toxic gas inhalation, lung infections (bacteria, viruses, pneumocystis, etc.), drowning, shock, sepsis, lung trauma [22], among which shock, external trauma and infection have been reported as the most common causes [23, 24]. The pathogenesis of ARDS basically includes three processes: the migration and aggregation of inflammatory cells, the release of inflammatory mediators, the damage of pulmonary capillaries and the increase in permeability [25]. As an acute inflammation, ARDS may have no obvious symptoms before the onset. Moreover, because it is mostly caused by some primary diseases, it is often concealed by the symptoms of primary diseases in the diagnosis, which leads to the aggravation of ARDS and even death of the patient [26].

European Society of Intensive Care Medicine (ESICM), the American Thoracic Society (ATS) and the Society of Critical Care Medicine (SCCM) jointly organized and revised the new ARDS diagnostic criteria—the Berlin Definition in 2012 [27]. In Berlin Definition, the gold standard for the diagnosis of ARDS is the imaging findings: there are plaque shadows in both lungs that cannot be fully explained by pleural effusion, atelectasis, or nodules on chest radiograph [28]. The chest CT examination is also known as the most accurate method of ARDS diagnosis, due to its higher sensitivity and accuracy [29]. However, for critically ill patients in ICU, especially patients with unstable hemodynamics or patients who are not suitable to be moved due to tracheal intubation, chest CT examination is rather limited. Although chest X-ray (CXR) can replace CT for bedside examination, it has low sensitivity and specificity [30]. X-ray superimposes the imaging information of the chest on a piece of film. Due to the influence of the heart, mediastinum and diaphragm, lung lesions may be hidden and cannot be displayed. In addition, mobile bedside radiographs have a low diagnostic sensitivity rate for lung bottom infiltration. Accumulating evidence has shown that the consistency of different observers on the presence of infiltration in the same chest radiograph is rather low [31]. The disadvantages of both CT and X-rays are high cost and radiation exposure, which makes chest CT and X-rays especially unsuitable for examinations of special populations such as pregnant women and young children.

In this research, we chose to use an accurate, non-invasive, real-time and more convenient method—LUC to check and evaluate the condition of ARDS patients who cannot move in the ICU. The cost of lung ultrasound is much lower than traditional CT methods due to equipment and principles. More importantly, bedside LUC has the advantages of higher feasibility, smaller instrument footprint and less time consuming compared to bedside CT or X-ray. LUC can reduce the handling and radiation exposure of critically ill patients, and can display the changes in lung morphology in real time. We believe that LUS is also an effective tool for distinguishing acute cardiogenic pulmonary edema and ARDS. LUS can identify ARDS early because its ultrasound abnormalities are earlier than changes in blood gas analysis PaO_2_/FiO_2_, and the ultrasound findings are highly consistent with the progression of lung injury and CT images. More importantly, by constructing a LUS scoring system, we can quickly evaluate the prognosis of extubation of ARDS patients with LUS detection, and design the corresponding treatment and nursing strategies, which is otherwise difficult to achieve with only CT or X-ray imaging results.

In recent years, LUS has become a simple and reliable method for the clinical diagnosis of lung diseases, especially in ICU [32]. LUS provides a lot of effective information for changes in lung morphology. Indirect signs through ultrasound, including abnormal pleural line, a disappearance of line, abnormal increase of B-line, and lung consolidation, can assist the doctors to evaluate the progression of lung diseases [33]. The LUS characteristics of ARDS mainly include lung consolidation with bronchial inflation, abnormal pleural lines, diffuse pulmonary edema and disappearance of A-line. When the three signs of pulmonary consolidation, diffuse pulmonary edema and abnormal pleural line are present at the same time, the sensitivity and specificity of LUS diagnosis are both quite high [13]. Therefore, as an emerging bedside imaging diagnostic technology, LUS has the advantages of non-invasive, simple, reproducible, and convenient for dynamic observation. Effective information shown by LUS is of great significance for the early diagnosis, dynamic evaluation and follow-up of a variety of lung diseases.

Interestingly, the results of LUS have been widely used to predict the outcome and prognosis of many lung diseases. For example, Wang et al. reported in 2016 that LUS could be used to predict the therapeutic effect of prone position on ARDS and the prognosis of ARDS patients [34]. Zhao et al. reported in 2015 that LUS images could be used to measure the EVLW of ARDS patients [35]. In this study, we tried to use the LUS score to predict the post-extubation distress in ARDS patients passing the SBT. We performed LUS examinations on ARDS patients before, at the end, and 4 h later, and determined whether the extubated patients can be successfully free of the ventilator after 48 h as outcomes. We found that patients with higher LUS scores tend to exhibit post-extubation distress. The LUS score examined at the end of SBT has the potential to predict the onset of post-extubation distress.

In fact, the clinical application of LUS also has some limitations. LUS is mainly applied to the lesions involving the pleura. The application of LUS in deep lung lesions that do not involve the pleura is limited. For example, the lung consolidation caused by tumors is often mixed with gas, which could hinder the propagation of sound waves, resulting in unclear LUS images or even undetectable reality. We will continue to pay attention to this issue in our future studies.

## Conclusion

In conclusion, we tried to illustrate the predictive effect of LUS scores measured at different time points on the outcome of ARDS patients 48 h after extubation in this study. We demonstrated that post-extubation distress correlated with higher LUS scores measured before SBT, at the end of 60-min SBT, and four hours after SBT. The LUS score measured before SBT and other predicted post-extubation distress assessments including APACHE II, SOFA, CPIS, EVLWI showed positive linear correlations. The LUS score examined at the end the 60-min SBT can be used to predict the incidence of post-extubation distress in ARDS patients.

## Data Availability

All data generated or analyzed during this study are included in this published article.

## Abbreviations

ARDS: Acute respiratory distress syndrome
SBT: Spontaneous breathing trial
LUS: Lung ultrasonography
